# Flexible migratory choices of Cory’s shearwaters are not driven by shifts in prevailing air currents

**DOI:** 10.1038/s41598-018-21608-2

**Published:** 2018-02-20

**Authors:** Gaia Dell’Ariccia, Simon Benhamou, Maria P. Dias, José P. Granadeiro, Joël Sudre, Paulo Catry, Francesco Bonadonna

**Affiliations:** 1 Behavioural Ecology Group, CEFE, CNRS, Montpellier, France; 2 Biodiversity Research Institute (IRBio) and Departament de Biologia Evolutiva, Ecologia i Ciències Ambientals, Universitat de Barcelona, Barcelona, Spain; 3 Marine and Environmental Sciences Centre (MARE), ISPA – Instituto Universitário, Lisbon, Portugal; 4 BirdLife International, Cambridge, UK; 5 Centro de Estudos do Ambiente e do Mar (CESAM), Faculdade de Ciências, Universidade de Lisboa, Lisbon, Portugal; 6 LEGOS-GRGS-UMR5566, Observatoire Midi-Pyrénées, Toulouse, France

## Abstract

Wind conditions strongly affect migratory costs and shape flyways and detours for many birds, especially soaring birds. However, whether winds also influence individual variability in migratory choices is an unexplored question. Cory’s shearwaters (*Calonectris borealis*) exhibit migratory flexibility, changing non-breeding destination across the Atlantic Ocean within and between years. Here, we investigated how wind dynamics affect the spatiotemporal migratory behaviour and whether they influence individual choices of non-breeding destination. We analysed 168 GLS tracks of migratory Cory’s shearwaters over five years in relation to concurrent wind data. We found no evidence for an association of the use of specific paths or destinations with particular wind conditions. Our results suggest that shearwaters deliberately choose their non-breeding destination, even when the choice entails longer distances and higher energetic costs for displacement due to unfavourable wind conditions en route. Favourable winds trigger migration only when directed towards specific areas but not to others. Despite their dependence on wind for dynamic soaring, Cory’s shearwaters show a high individuality in migratory behaviour that cannot be explained by individual birds encountering different meteorological conditions at departure or during migratory movements.

## Introduction

Optimal migration theory predicts that wind conditions are one of the most important factors affecting avian migratory behaviour, deeply influencing the energetic costs of displacements. Wind is thus an important selective agent in the evolution of migration patterns. Migrating birds are expected to travel routes where they can benefit from frequently occurring favourable winds and selection pressure should favour strategies in which individuals undertake migration under advantageous atmospheric conditions e.g.^1–3^. In fact, different bird species are able to sense and predict wind changes and shape their migration in order to take advantage of the most favourable winds, with departures often being determined by the onset of favourable wind conditions e.g.^4–6^.

Soaring birds are particularly sensitive to wind patterns. An interesting case is that of petrels (petrels, shearwaters and albatrosses, Order Procellariiformes). These seabirds spend most of their lives flying over the vast ocean; they are tied to small islands only for breeding. Each year they complete astonishing migrations that can be as long as 70 000 km crossing the oceans of both hemispheres^7–10^. To complete these flights, petrels have evolved a low energetic cost flight mode, the dynamic soaring, which enables them to extract the energy necessary for flying directly from the wind over the waves and the ocean surface^11,12^. They live in an environment where winds are stronger and more constant than on land, and the conditions they encounter at departure and during migration may have a deep influence on the cost of migration^3^ and references therein. Indeed, prevailing wind patterns are believed to have an important role in shaping general migratory timings, paths, and detours of shearwaters^8,9,13,14^.

The Cory’s shearwater (*Calonectris borealis*) is a particularly interesting case because of its flexibility in migratory behaviour. This species is a medium-sized petrel that breeds on small islands of the North Atlantic Ocean and winters in four broad areas: the North-West Atlantic and the Canary Current area, in the northern hemisphere, and the Brazilian Current area-Central South Atlantic and the Benguela-Agulhas Current area, in the southern hemisphere^7^. Contrary to most other petrels, this species shows the remarkable ability to change non-breeding destination both within and between years, switching between areas located more than 7000 km apart from each other. There is no evidence that this flexibility in individual choice is influenced by age, sex or individual quality, nor by oceanographic conditions (e.g. sea surface temperature and chlorophyll α concentration) of the non-breeding areas^7^. Carrying over from the preceding breeding season tends to affect migratory behaviour: failed male breeders tend to skip migration, remaining sedentary in the Canary Current area for the whole winter^15^, and breeding experience appears to affect the number of visited non-breeding sites^16^, but no other influences have been reported on the choice of different non-breeding areas.

In this study, we analysed the at-sea behaviour of Cory’s shearwaters recorded by geolocators during their long-distance journeys across the Atlantic Ocean, relating their post-breeding migratory paths and choices of non-breeding destination to local wind conditions. Prevailing wind patterns over the ocean shape the general migration routes of this species^13,14^, but whether they also have an influence on their individual flexibility and choices of non-breeding areas is a question that has never been addressed.

## Results

**Figure 1:**
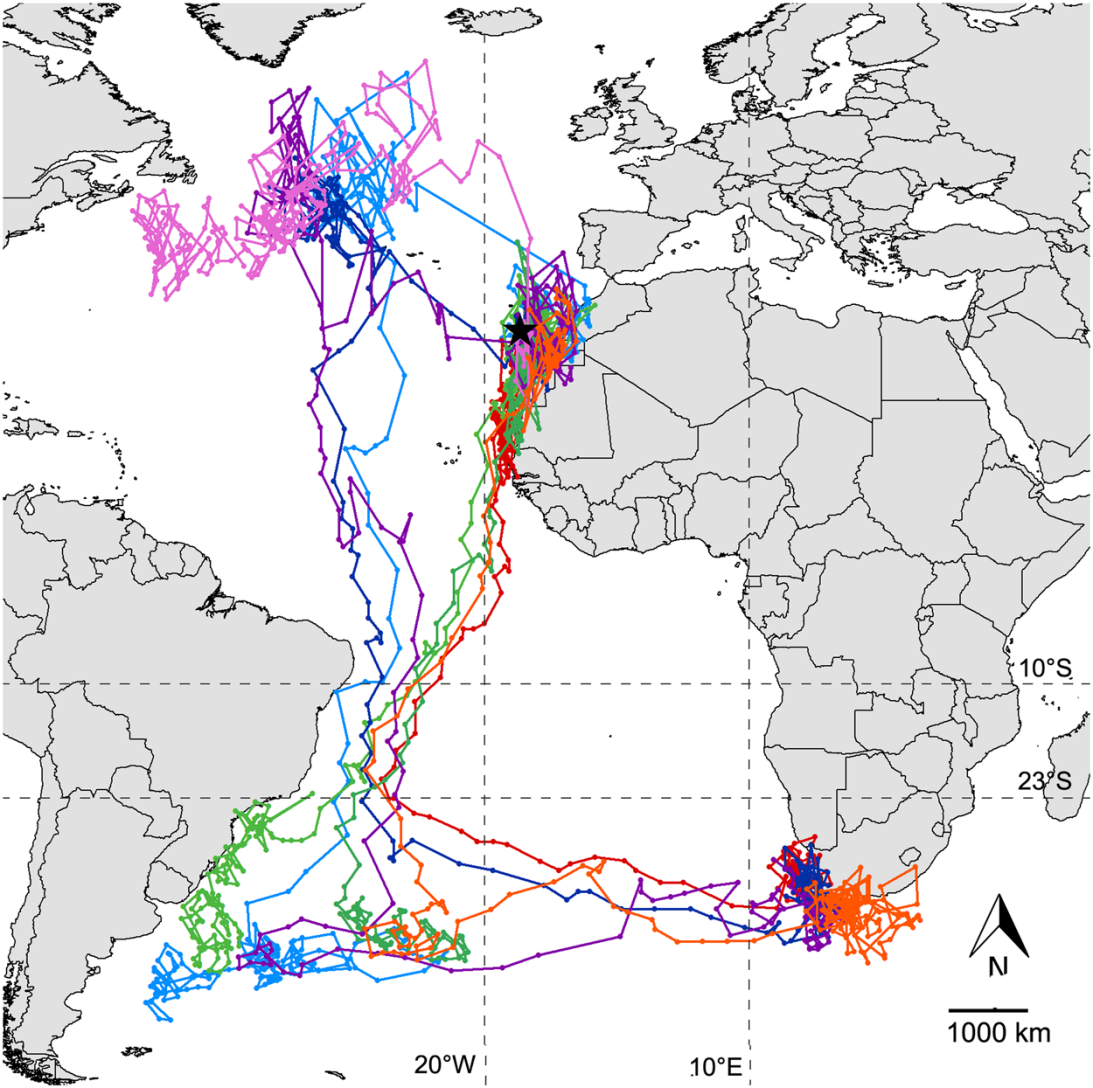
Main migratory routes and non-breeding areas. The figure illustrates one representative raw GLS track per destination category. The black star indicates the colony. Pink: a track to North-West Atlantic (NWA); dark blue: a track to NWA later resuming migration to South Africa (SAF); light blue: a track to NWA later resuming migration to South America (SAM); purple: a track to NWA later resuming migration to SAM and then to SAF; green: two tracks to SAM (to illustrate the wide range of this area. See also Dias *et al*.^7^); orange: a track to SAM later resuming migration to SAF; red: a direct track to SAF. Dashed lines mark latitudes and longitudes references for track segments before the split of destination to SAM or SAF and included between SAM and SAF, respectively (see main text). This map was generated using ArcGIS 9.3.1 (www.esri.com).

We analysed 168 post-breeding migratory tracks. For the analyses concerning departure we considered only 154 tracks, 22 of which corresponded to migration towards the North-West Atlantic (NWA) and 132 corresponded to migration towards the South Atlantic (SA, See Fig. 1 for an example of tracks and regions), because ten tracks heading to North-West Atlantic and four heading to South Atlantic started during the autumn equinox period when geolocation data are unreliable. Birds migrating towards North-West Atlantic started their migration consistently earlier (29.5 ± 10.8 days; hereafter all values provided as as x ± y corresponds to mean ± standard error) than birds heading towards South Atlantic, with no significant differences among years (GLM, effect of migratory destination: *F*_*4*,*144*_ = 8.59, *p* = 0.004; effect of year: *F*_4,144_ = 0.82, *p* = 0.51).

**Figure 2:**
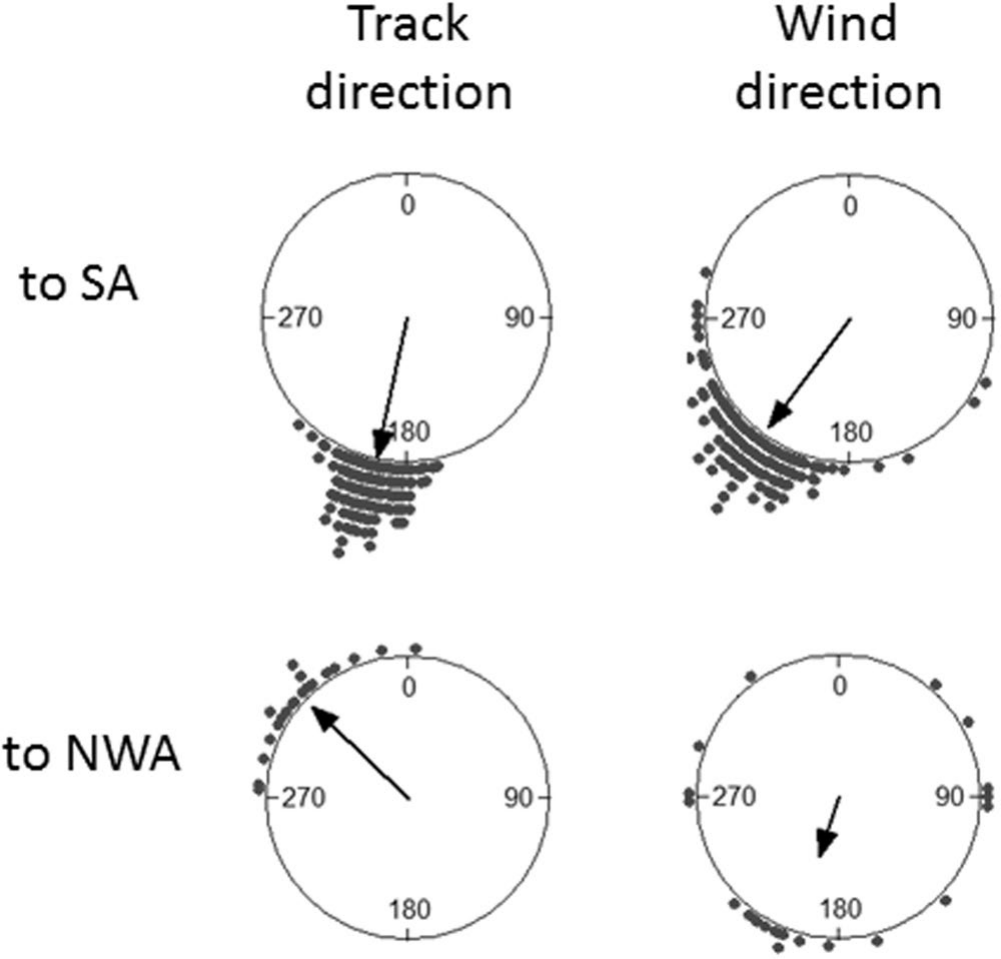
Average track direction between the target location and the following one calculated on the three days following departure and the corresponding wind direction experienced, by shearwaters heading to South Atlantic (SA) and North-West Atlantic (NWA). Arrows indicate mean vectors. To SA track vector: Φ = 192.2°, *r* = 0.986, Rayleigh test *p* < 0.0001; to NWA track vector: Φ = 313.5, *r* = 0.933, Rayleigh test *p* < 0.0001; to SA wind vector: Φ = 217.1°, *r* = 0.927, Rayleigh test *p* < 0.0001; to NWA wind vector: Φ = 198°, *r* = 0.429, Rayleigh test *p* = 0.016.

**Figure 3:**
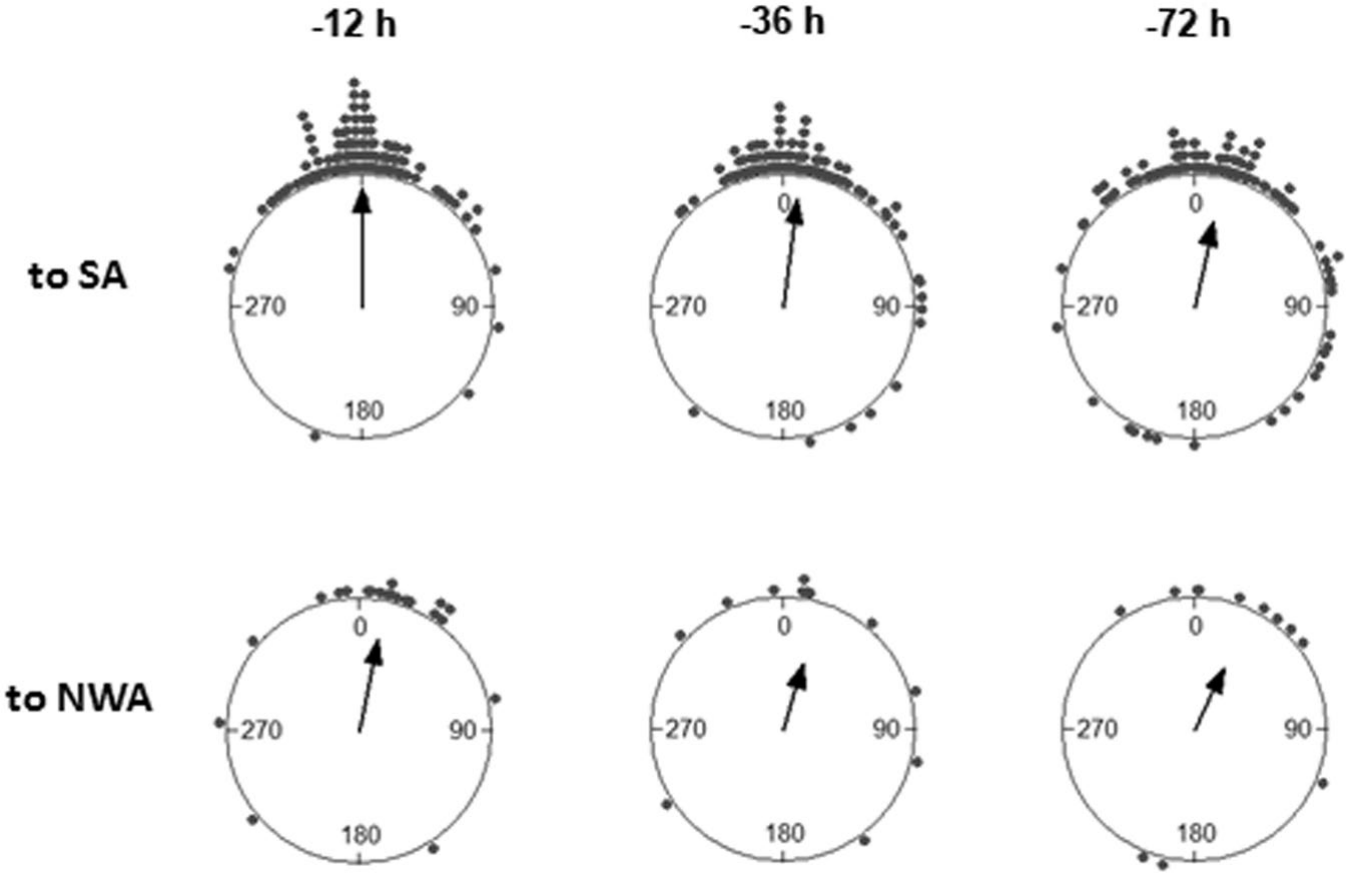
Difference in wind direction experienced by birds 12, 36 and 72 hours before departure with respect to the wind experienced at departure (corresponding to 0), for birds heading to South Atlantic (SA, upper part) and to North-West Atlantic (NWA, lower part). Arrows indicate mean vectors.

When departing from the colony area, shearwaters flying to South Atlantic or to North-West Atlantic oriented to different mean directions (Watson-Williams F-test: *F* = 1405.12, *n*_1_ = 132, *n*_2_ = 22, *p* < 0.0001; Fig. 2). At this early stage of migration, both groups of birds experienced winds whose mean direction did not differ significantly (Watson-Williams F-test: *F* = 3.639, *n*_1_ = 132, *n*_2_ = 22, *p* = 0.058. Figure 2), but were significantly different in terms of dispersion (non-parametric dispersion test: *U* = 799.5, *p* < 0.001). Thus, when leaving the colony area, birds migrating to South Atlantic flew on average within 25° of the wind direction, i.e. with a tailwind, whereas most of birds migrating to North-West Atlantic flew against the wind. The analysis of wind directions revealed that, for birds migrating to South Atlantic, there was a high autocorrelation in the experienced wind directions in the 12 h lag before departure for migration (V test: *V* = 0.895, *n* = 120, *p* < 0.0001). Thus, at 12 h before departure, shearwaters migrating to South Atlantic had a wind direction similar to the wind direction at departure. However, for these birds, the wind direction at 36 and 72 h before departure was significantly more variable (paired Wilcoxon-Mann-Whitney test for angular dispersion: *T* = 1488, *p* = 0.008 and *T* = 1082, *p* < 0.0001, respectively; Fig. 3), indicating winds coming from different directions at this moment. For birds migrating to North-West Atlantic, there was also some autocorrelation in the experienced wind directions in the same 12 h lag, although smaller than for birds migrating to South Atlantic (V test: *V* = 0.684, *n* = 21, *p* < 0.0001), but the wind scatter at 12 h before departure was not significantly different to those observed 36 h or 72 h before departure (paired non-parametric dispersion test: *T* = 19, *p* = 0.13 and *T* = 20, *p* = 0.15, respectively; Fig. 3). This suggests a change in wind conditions prior to departure and that shearwaters waited for favourable winds when migrating to South Atlantic but not when migrating to North-West Atlantic.

Considering the migration segment between the colony and the first non-breeding area reached by the birds (excluding possible intermediate stop-overs), intra-track correlations between flight and wind velocity vectors (i.e. simultaneously taking into account both direction and speed) were statistically significant (exact permutation test *p* < 0.05) for all tracks. This result indicates that the differences between the two vectors (expressed in terms of absolute values) tended to remain constant during this segment. The average intra-track correlations between flight and wind velocities for birds heading to North-West Atlantic were however significantly higher than for birds flying to South Atlantic (NWA: *ρ*_*v*_^2^ = 1.20 ± 0.62; SA: *ρ*_*v*_^2^ = 0.62 ± 0.02; Wilcoxon-Mann-Whitney for independent samples: *U* = 2090, *p* < 0.0001), indicating a more stable relationship between flight and wind vectors for these birds. The analysis of the mean tailwind component (TWC, averaged within each track) showed that birds heading to South Atlantic benefitted from a facilitating effect of the wind (TWC = 1.64 ± 0.09 m·s^−1^), while birds heading to North-West Atlantic flew against the wind (TWC = −0.20 ± 0.47 m·s^−1^). The difference between the two groups was significant (t-test: *t* = 3.85, df = 19.4 *p* = 0.001).

**Figure 4:**
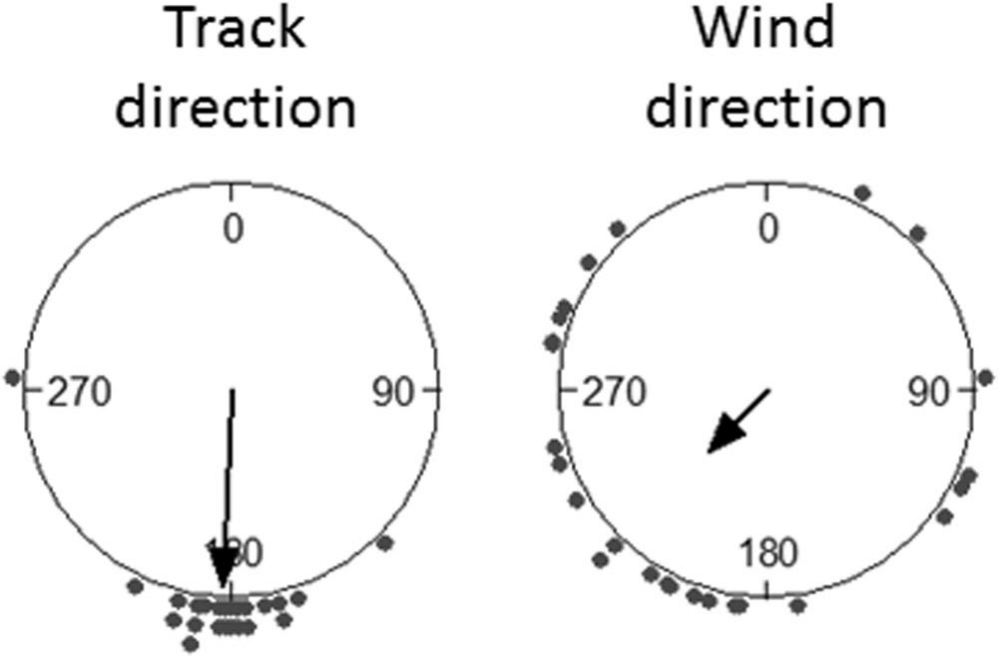
Average track and wind directions on birds departing from North-West Atlantic. Arrows indicate mean vectors. Track: Φ = 182.8°, *r* = 0.94, Rayleigh test *p* < 0.0001; wind: Φ = 223.2°, *r* = 0.4, Rayleigh test *p* = 0.016.

Some birds (*n* = 24) that flew to North-West Atlantic remained there only for a period and then left this area to migrate to South Atlantic. At departure from North-West Atlantic, birds were all well oriented southwards and, on average, experienced a favourable wind direction, even if winds were quite dispersed (Fig. 4). In the migration segment from North-West Atlantic to the following non-breeding area in South Atlantic, scores of TWC indicated a facilitating effect of the wind (TWC = 1.15 ± 0.16 m·s^−1^), although significantly less favourable than that of birds heading to South Atlantic directly from the colony (t-test: *t* = 2.691, df = 44.1, *p* = 0.01). The mean intra-track vector correlation coefficient between flight and wind velocities during their southwards migration (*ρ*_*v*_^2^ = 0.54 ± 0.04) was not significantly different from the mean correlation coefficient for birds departing directly from colony to South Atlantic (t-test: *t* = 1.77, df = 35.5, *p* = 0.085), indicating that the relationship between flight and wind velocities showed the same degree of stability in both cases.

**Figure 5:**
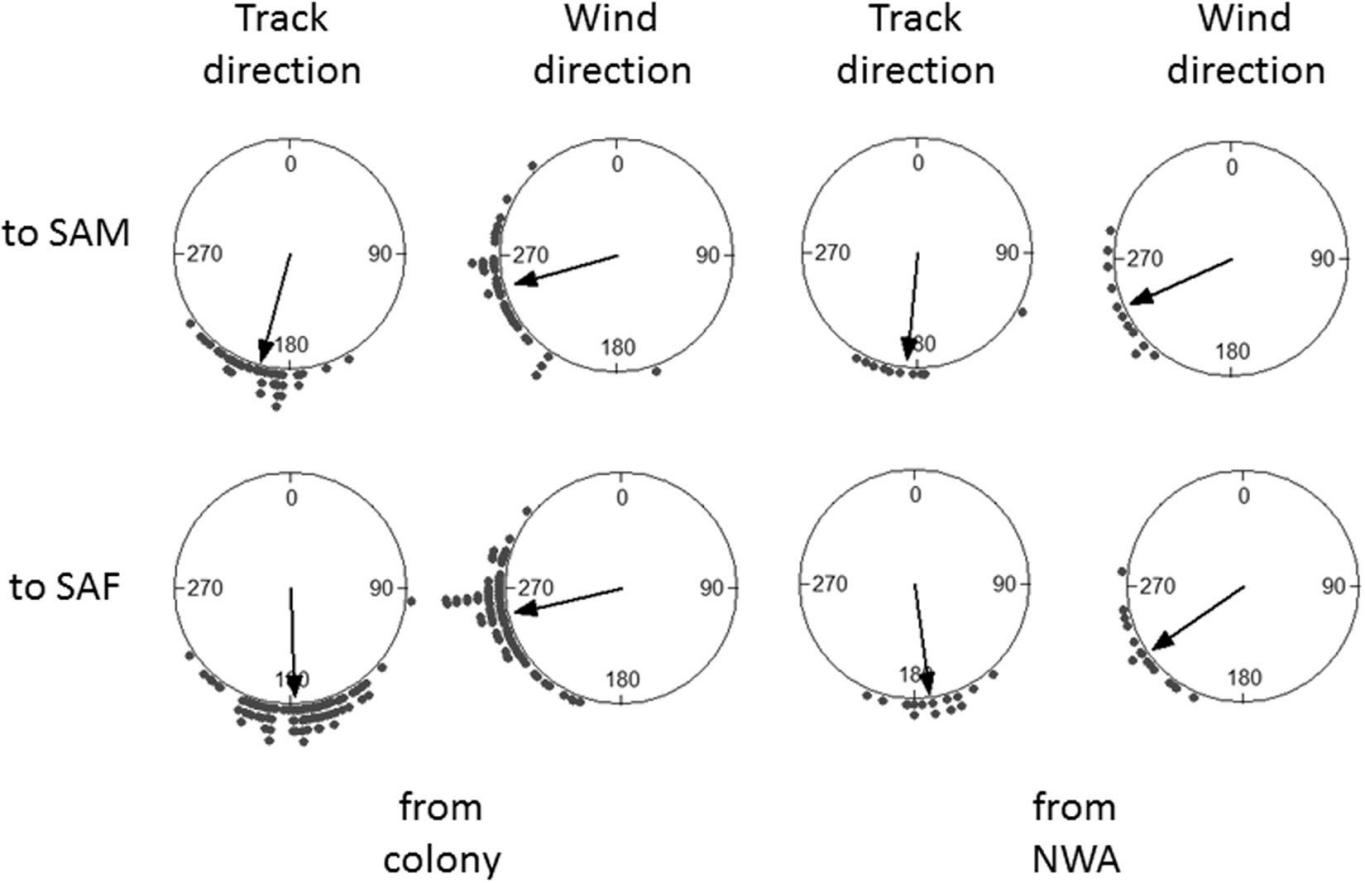
Wind and track average directions in the track portion included between 10°S and the Tropic of Capricorn for individuals migrating from the colony (left) and from North-West Atlantic (NWA, right) and directed to South America (SAM, up) and South Africa (SAF, low).

Birds migrating to South Atlantic (132 directly from the colony area and 24 via North-West Atlantic) headed towards the southwest Atlantic (Fig. 1). After crossing the Tropic of Capricorn (ca. 23°S), some of them (*n* = 62, 52 directly from the colony and 10 via North-West Atlantic) stopped for the non-breeding period in the waters off South America (SAM), in the Brazilian Current area and central south Atlantic, while others (*n* = 94, 80 directly from the colony and 14 via North-West Atlantic) continued their trip directly to South Africa (SAF), to spend the non-breeding period in the Benguela-Agulhas current areas (Fig. 1). To determine whether the wind conditions influenced the birds’ decision to stop in South America or continue their migration directly to South Africa, we specifically investigated the relationship between the flight and wind conditions experienced in the area crossed just before the divergence of tracks, between 10°S latitude and the Tropic of Capricorn (Fig. 1). We found no significant differences in average intra-track vector correlation coefficients between flight and wind velocities (Wilcoxon-Mann-Whitney test for independent samples: *p* > 0.05 for all comparisons) between birds stopping in South America (coming directly from colony: *ρ*_*v*_^2^ = 0.93 ± 0.07, or from NWA: *ρ*_*v*_^2^ = 0.87 ± 0.12) or those flying to South Africa (from colony: *ρ*_*v*_^2^ = 0.92 ± 0.05, or from NWA *ρ*_*v*_^2^ = 1.1 ± 0.1). Nevertheless, for shearwaters coming directly from the colony, the tailwind component when crossing the 10°S-Tropic of Capricorn area was significantly higher for those stopping in South America (TWC = 2.7 ± 0.27 m·s^−1^) than for those directly flying to South Africa without stopping (TWC = 1.09 ± 0.22 m·s^−1^; t-test: *t* = 4.7, df = 96.7, *p* < 0.001), whereas for shearwaters coming from North-West Atlantic, the difference was not significant (2.92 ± 0.49 vs. 2.87 ± 0.36 m·s^−1^; t-test: *t* = 0.08, df = 19.6, *p* = 0.93). Looking at the average flight and wind directions in this portion of path, it appeared that this difference is mainly due to a different orientation of birds, while encountering similar wind conditions (Fig. 5).

Finally, 40 of the shearwaters that stopped off South America resumed their migration to South Africa after a variable time spent in South America waters. In the segment of track included between the two non-breeding areas (i.e. between longitudes 20°W and 10°E; Fig. 1), these birds benefited from favourable wind conditions (TWC = 2.13 ± 0.4 m·s^−1^), yet not significantly different from those encountered by birds that directly migrated toward South Africa without stopping (TWC = 1.66 ± 0.20 m·s^−1^; t-test: *t* = −1.05, df = 32.3, *p* = 0.3). For these two groups of birds, the difference between vector correlations between flight and wind velocities was also non-significant (*ρ*_*v*_^2^ = 0.83 ± 0.06 vs. *ρ*_*v*_^2^ = 0.90 ± 0.03; t-test: *t* = −1.06, df = 34.9, *p* = 0.3).

## Discussion

Cory’s shearwaters deliberately choose their non-breeding destination, even when this implies flying with headwinds. While birds generally wait for favourable winds to start migration^1,4,6^, we show that a change in wind direction appeared to trigger the departure only for shearwaters migrating directly to the southern hemisphere, but not for birds heading to the North Atlantic. Our analyses showed that Cory’s shearwaters did not only fly following favourable winds, and that wind conditions alone did not determine the choice of the non-breeding ground. Vectorial correlation analyses showed that during migration the birds tended to maintain a stable relationship between their flight and the wind conditions. Both at departure from colony and during the whole migration from the colony to the first visited non-breeding area, only shearwaters heading southwards benefited from a facilitating effect of the wind (i.e. positive values of the tailwind component), whereas birds migrating to North-West Atlantic tended to experience headwinds. In addition, birds that resumed their migration towards the South Atlantic after migrating to the North-West Atlantic, tended to benefit from less favourable wind conditions than the birds migrating directly southwards from the colony. Similarly, shearwaters undertaking a longer migration directly to waters off South Africa tended to experience slightly less favourable wind conditions than shearwaters that stopped off South America. The lower scores in tailwind component reached by the birds travelling to South Africa, however, were due more to the different direction of birds’ migratory track than to different wind conditions, highlighting that shearwaters chose their non-breeding destination independently of wind conditions encountered en route. Finally, when crossing eastwards the south Atlantic between South America and South Africa, all birds experienced similar wind conditions, irrespective of whether they previously stopped off South America or not.

Thus, Cory’s shearwaters’ choices of non-breeding areas seem to be constrained by factors other than wind conditions. One possibility is that they may behave according to the ideal free distribution theory to maximise the availability of food resources per individual^17,18^ across different non-breeding areas. This theory assumes that each individual selects the area best suited to its survival, and that area suitability is inversely proportional to the number of competitors exploiting that area. Consequently, once a critical density of competitors has been reached, some birds in the area, as well as newly arriving individuals, could improve their chances of success by moving to an area of higher suitability, i.e. a less rich or more distant food source but where less competition occurs, in order to obtain the same food intake rate^17,18^.

The seemingly energetic waste by birds flying to North-West Atlantic instead of remaining around the colony in the Canary Current area before southward migration may be explained in the light of this hypothesis. The Canary Current area is a highly profitable foraging ground, where a high number of individuals from different colonies concentrate after the breeding season to forage before migration^7,19–21^ and to wait for the stopping of the westerly winds of the African Monsoon in order to cross the Intertropical Convergence Zone of the Atlantic^14^. A number of individuals even skip migration, spending the whole winter in this area e.g.^7^. The very high density of individuals in the Canary Current area may make the competition too strong for some birds, inducing them to move to another area, spending an energetic cost for displacement, which may be compensated by reduced competition. This hypothesis is strengthened by the observation that individuals migrating to North-West Atlantic leave the colony and the Canary Current area earlier that those that migrate directly southwards, but the subsequent southwards migration takes place at the same period of individuals migrating directly from the colony^19^. Our results showed that migrating to the North-West Atlantic involves a higher energetic flying cost due to less favourable winds, not only from the colony to the North-West Atlantic, but also from the North-West Atlantic to the South-Atlantic, when compared to migrating directly from the colony to the South-Atlantic. We speculate that a higher ratio of food availability over competitor density might compensate for this higher cost, making this option more profitable than staying around the colony in the Canary Current area. When heading towards North-West Atlantic, our tracked birds did not select a favourable wind at departure to maximize initial wind assistance, as other birds do when migrating^4,6^, this study. The North Atlantic gyre results in highly predictable winds that are almost never favourable for flying towards the North-West Atlantic^3^. Shearwaters might thus have learnt that, when deciding to travel to North-West Atlantic, it is useless to wait for a highly improbable, favourable wind to depart. To support this hypothesis, it would be necessary to test the relation between this peculiar migratory behaviour and the birds’ experience.

The ideal free distribution theory may also explain the choices displayed by shearwaters that move to the South Atlantic. The Brazilian Current area is highly productive and frequented by large numbers of several petrel species which spend the non-breeding season there e.g.^8,20,22^. This is also a closer non-breeding area than South Africa, resulting in a lower migratory energetic cost for shearwaters stopping in South America. However, increasing numbers of birds in the area can lead to an increasing competition for food resources, and we hypothesize that a fraction of Cory’s shearwaters may decide to continue their trip directly towards South Africa (or to resume migration towards South Africa after a stop off South America). Despite a longer trip, the energetic balance might be positive as the ratio of available resources over competitor density might be more favourable. Cory’s shearwaters stopping off South America, on average, start their southward migration about four weeks earlier than birds directly migrating to areas off South Africa (average departure date: October 22^nd^ vs. November 13^th^, respectively). Despite a large variability in the dates of departure for shearwaters that stop off South America, there is a clear tendency for most early birds to spend the non-breeding season in South America waters. In contrast, most late birds tend to stop off South Africa, supporting the idea that competition for food resources may increase off South America over time, due to increasing numbers of birds foraging in the area. As alternative hypothesis, birds stopping off South America maybe in poor physical conditions, needing to fuel-up again before resuming their migration to South Africa or avoiding flying further to prevent supplemental displacement costs. Both hypotheses need to be tested. It would be interesting to investigate whether there is a relationship between breeding parameters, e.g. breeding success, the fledging date, and the date of departure, which might influence the migratory strategy, and the final non-breeding destination; or to explore whether the body conditions of individuals at the end of the reproductive season may affect migration decision-making, affecting their abilities to cope with foraging competition, migration distances and with more or less favourable wind conditions.

The potential ability of decision-making highlighted in our study implies that Cory’s shearwaters have some knowledge of the potential spatial distribution of resources, possibly based on experience acquired in previous years. Further investigations are required to test this hypothesis. However, previous studies comparing the migratory behaviour of adult and juvenile procellariforms, or of other seabirds, suggest that the pre-breeding years spent at sea may serve to gather information on the spatial distribution of resources at sea^23–25^. According to the “exploration-refinement hypothesis”, pre-breeding large-scale exploratory movements would allow individuals to refine their migratory routes and behaviour through learning^23^. The knowledge of alternative non-breeding grounds and their average relative profitability by long-living migrating birds fits well in this framework.

In conclusion, despite the dependence of Cory’s shearwaters on wind for travelling, oceanic wind patterns do not seem to be responsible for individual variability in migratory behaviour in this species. Cory’s shearwaters have the ability to deliberately choose alternative non-breeding areas, even when the choice entails more energetically expensive flying routes with less favourable winds and longer distances.

## Methods

We analysed a dataset of 168 migratory tracks from 101 breeding adult Cory’s shearwaters from Selvagem Grande Island (Portugal: 30°02′ N; 15°52′ W). The tracks were recorded by leg-mounted geolocators (mk 7 model from British Antarctic Survey, Cambridge; mass approx. 3.6 g) for five consecutive years, from the autumn migration in 2006 to the end of the spring migration of 2011^7,26^. For 58 individuals, a single migratory track was recorded, whereas for 23, 16 and four of them, two, three and four migratory tracks, respectively, were recorded. As most of the individuals were recorded only once, and the wind conditions and behaviour varied quite largely between years so that some averaging across years would have been meaningless, we considered the 168 migratory tracks as statistically independent, even when they were performed by the same individuals. Geolocators were deployed on birds at the end of the breeding season and collected at the beginning of the following breeding season. They provided two locations per day based on light levels, with an accuracy of approximately 200 km^27^. Light data were analysed using TransEdit (to check for integrity of light curves and to fit dawn and dusk times) and Birdtrack software (to estimate the latitude from day length and longitude from the time of local mid-day relative to Greenwich Mean Time) for more details, see^7^.

Because we aimed to understand the influence of winds on the choice of non-breeding grounds, we focused our analysis on the post-breeding migration only, ending the tracks at the arrival at the last visited non-breeding area. In addition, the pre-breeding migration of many Cory’s shearwaters coincided with spring equinox, during which geolocation data are unreliable. We obtained local wind conditions using the NOAA database, which provides daily sea surface wind velocity (i.e. speed and direction) fields, on a global ocean 0.25-degree grid (https://www.ncdc.noaa.gov/data-access/marineocean-data/blended-global/blended-sea-winds). Using linear spatio-temporal interpolation, we associated local wind conditions to each GLS location.

For each location of all tracks, we computed the Residence Time (RT)^28^ as the time spent within 300 km of this location, using a Mercator projection. During migration, birds are expected to perform fast, directional movements, which are associated with low values of RT, whereas stop-overs or establishment in non-breeding grounds is characterised by higher RT values. We thus identified the departure from the colony or from a non-breeding area and the arrival at non-breeding area(s) based on the onset or interruption of a clearly directional movement associated with RT values that decreased below 3 days and increased over 6 days, respectively. Locations with RT < 3 days were assumed to correspond to migratory movements and locations with RT > 6 days were assumed to correspond to stops in non-breeding areas. Ambiguous locations with RT values included between 3 and 6 days were excluded from the analyses. This is a more conservative choice than previous studies that had threshold between flight and stop at 4 days at 200 km scale with no intermediate level e.g.^7^, allowing us to be more confident when attributing the flight/non-breeding stop category to locations in our analyses.

To investigate the relationship between the departing directions of birds with respect to local wind condition, we compared wind and flight directions at the departing location between birds heading towards the North-West Atlantic (NWA) and the South Atlantic (SA) using the Watson-William F-test^29^. We computed individual flight directions as straight-line directions between two consecutive GLS locations for 3 days starting from the departing location (identified based on RT time as described above). To smooth noise due to low resolution of GLS locations, we then considered the average flight and wind (weighted for wind intensity) directions for these first 3 days following departure. To test whether migration departure was triggered by a change in wind conditions, we analysed the differences in direction between the wind experienced by shearwaters heading towards North-West Atlantic and South Atlantic at departure and 12, 36 and 72 hours before departure, using the Wilcoxon-Mann-Whitney test applied to angular deviations with respect to wind direction at departure See^29^ p.125, and adapted to paired data. We analysed the difference in departure dates for birds heading to different hemispheres using a general linear model using date as a dependent variable and zone and factor(year) as covariates.

We investigated the relationship between migratory tracks and concurrent winds taking into account the vectorial nature of these two parameters, i.e. they are described by both magnitude (wind or flight speed) and direction. The track vector associated to each location was calculated as the flight velocity (speed and direction) between one GLS location and the following one, representing the bird movement; wind vectors were calculated from the NOAA database (see above). We calculated the squared vector correlation ρ_*v*_^2^ (i.e. simultaneously taking into account direction and magnitude of the variables) between track and wind vectors^30,31^ symmetrised (i.e. the differences between the two directions were expressed in terms of absolute rather than arithmetic values) to consider wind directions irrespective of the side (left or right) of the bird. It is worth noting that ρ_v_^2^ ranges between 0 and 2 for vectorial correlations in 2D space see^31^, and that a high ρ_v_^2^ value means that the relationship between flight and wind vectors remained stable during migration, although the two vectors can be quite dissimilar in magnitude and/or direction. A single ρ_v_^2^ value was computed for each migratory track (the whole post-breeding migration, from colony to the last visited non-breeding area) or migratory segment (the segment of migration included between two specific locations such as, e.g. the departure location and the location of arrival at the first non-breeding area, or specific longitudes or latitudes to analyse migratory portions of particular interest in detail. See below for details on the segment limits in each analysis). We calculated significance for this intra-track ρ_v_^2^ value by a permutation test, by which we looked at where the observed ρ_v_^2^ value lied in the distribution of possible ρ_v_^2^ values obtained by multiple permutations of the order of one of the two variables assumed to be correlated. In addition, to determine whether the wind conditions were globally favourable or not, we computed the tailwind component (TWC)^4^ as *S*_*W*_cos(φ_*F*_-φ_*W*_), where *S*_*W*_ is the local wind speed, and φ_*F*_ and φ_*W*_ are the local flight and wind directions, respectively. This index of the facilitating effect of the wind was only used so far to explore vanishing bearings at departure for migration. We applied it here for the first time to whole migratory tracks from breeding to non-breeding grounds to understand the ecological constraints that regulate distribution and behaviour of a dynamic-soaring species. TWC values were computed at each migration location for which *S*_*W*_ was higher than 2 m/s and RT was lower than 3 days (i.e. we excluded stop-overs), and then averaged for the whole migratory track or migratory segment of interest of each bird. Based on mean TWC and on the vector correlations, we compared birds heading to North-West Atlantic and to South Atlantic, in their migratory segment from the departure location from the colony to the arrival location at their first non-breeding site. Similarly, we compared birds heading to the southern hemisphere when departing either from the colony or from the North-West Atlantic. Once in the southern hemisphere, shearwaters followed a narrow corridor on the southwest Atlantic and, after crossing the Tropic of Capricorn (ca. 23°S), some of the birds stopped in the waters off the Brazilian coast, while others continued their trip without stopping up to the South African waters (Fig. 1). Therefore, to explore possible differences in the facilitating effect of the wind that may account for birds to continue their migratory trip up to South Africa (SAF) or stop to South America (SAM), we specifically analysed the migratory segment included between latitude 10°S and the Tropic of Capricorn, i.e. just before the divergence of destination. Also, we calculated the averaged TWC and vector correlation for the migratory segment lying between longitudes 20°W and 10°E, i.e. the part of track between South America and South Africa (Fig. 1), to compare these variables for birds which stopped at South America and then resumed their trip to South Africa and those that flew directly to South Africa.

 RT, TWC, flight vectors and vector correlations were computed from raw location and wind data using home-made programs written in Pascal. Statistical tests were performed with R (v.3.3.1)^32^ and the package ‘plotrix’ for linear variables, or with Oriana (v.4.02, Kovach Computing Services) for circular ones.

### Data availability

Data are deposited in the BirdLife Seabird database.
